# Giant FAZ10 is required for flagellum attachment zone stabilization and furrow positioning in *Trypanosoma brucei*

**DOI:** 10.1242/jcs.194308

**Published:** 2017-03-15

**Authors:** Bernardo P. Moreira, Carol K. Fonseca, Tansy C. Hammarton, Munira M. A. Baqui

**Affiliations:** 1 Department of Cellular and Molecular Biology and Pathogenic Bioagents, Ribeirão Preto Medical School, University of São Paulo, Ribeirão Preto 14049-900, Brazil; 2 Institute of Infection, Immunity and Inflammation, College of Medical, Veterinary and Life Sciences, University of Glasgow, Glasgow G12 8QQ, UK

**Keywords:** Cytoskeleton, Giant protein, FAZ10, FAZ, Nucleus positioning, Kinetoplast positioning, Cytokinesis

## Abstract

The flagellum and flagellum attachment zone (FAZ) are important cytoskeletal structures in trypanosomatids, being required for motility, cell division and cell morphogenesis. Trypanosomatid cytoskeletons contain abundant high molecular mass proteins (HMMPs), but many of their biological functions are still unclear. Here, we report the characterization of the giant FAZ protein, FAZ10, in *Trypanosoma brucei*, which, using immunoelectron microscopy, we show localizes to the intermembrane staples in the FAZ intracellular domain. Our data show that FAZ10 is a giant cytoskeletal protein essential for normal growth and morphology in both procyclic and bloodstream parasite life cycle stages, with its depletion leading to defects in cell morphogenesis, flagellum attachment, and kinetoplast and nucleus positioning. We show that the flagellum attachment defects are probably brought about by reduced tethering of the proximal domain of the paraflagellar rod to the FAZ filament. Further, FAZ10 depletion also reduces abundance of FAZ flagellum domain protein, ClpGM6. Moreover, ablation of FAZ10 impaired the timing and placement of the cleavage furrow during cytokinesis, resulting in premature or asymmetrical cell division.

## Abstract (precis)

**Summary:** Functional characterization of the *T. brucei* giant protein FAZ10 reveals it has roles in flagellar attachment, nucleus and kinetoplast positioning and, uniquely for a FAZ protein, a direct role in cytokinesis.

## INTRODUCTION

*Trypanosoma brucei* is the causative agent of human African trypanosomiasis and belongs to the Trypanosomatidae family. Trypanosomatids have developed unique characteristics, such as the cytoskeleton, that differentiate these organisms from other eukaryotes. The cytoskeleton is a well-defined component of the cell with two main structures: a subpellicular corset of microtubules that defines cell shape and size, and a flagellum that is crucial for motility and cell division (Hemphill et al., 1991). The cytoskeleton undergoes rearrangement during cell morphogenesis and division, making this structure essential for trypanosome biology (Sherwin and Gull, 1989). The flagellum, which emerges from a small invagination of the plasma membrane named the flagellar pocket, runs attached to the cell body along its length and consists of the axoneme and the paraflagellar rod (PFR), which are essential structures for a wide range of cellular processes, including cell motility, morphogenesis, division and infectivity (Bastin et al., 1998; Gull, 1999; Kohl et al., 2003; Broadhead et al., 2006; Emmer et al., 2010). A flagellum attachment zone (FAZ) is primarily responsible for flagellar attachment and stabilization. Initially, the FAZ was shown to be a cytoskeletal bundle of fibrillar links resembling a desmosome-like structure (Hemphill et al., 1991). Now, it is known that it is a large and complex interconnected set of fibres, filaments and junctional complexes that link the flagellum to the cell body, and that comprises five main domains (FAZ flagellum domain, FAZ intracellular domain, FAZ filament domain, microtubule quartet domain and microtubule quartet-FAZ linker domain) that can be subdivided into eight overlapping zones (Sunter and Gull, 2016). It is important to identify and characterize new components of each of these zones in order to understand FAZ biogenesis and function.

It has been suggested that depending on the location of a given FAZ protein, the RNAi phenotype can often be predicted (Sunter and Gull, 2016). Knockdown of some proteins located at the FAZ flagellum domain results in FAZ shortening and morphological changes. For instance, knockdown of Calpain-like protein GM6 (ClpGM6), initially described by Müller et al. (1992), leads to repositioning of the kinetoplast, basal body, Golgi and flagellar pocket, and to the formation of an epimastigote-like morphology, characterized by the repositioning of the kinetoplast to anterior of the nucleus (Hayes et al., 2014). Similar morphogenetic defects were also observed after knockdown of FLAM3, another FAZ flagellum domain protein (Sunter et al., 2015b). At the cell body side, the FAZ filament domain comprises several proteins, most importantly FAZ1, CC2D, FAZ2 and FAZ9. FAZ1 knockdown was reported to lead to flagellum attachment defects, and although the FAZ was still being formed, cells without FAZ1 showed unequal segregation of nuclei and kinetoplasts during cytokinesis (Vaughan et al., 2008). In contrast, CC2D was reported to be present in the FAZ and basal body of *T. brucei*, with its knockdown leading to the absence of the FAZ in daughter cells with major effects on the subpellicular cytoskeleton architecture, flagellum detachment, shorter cell body and cell death (Zhou et al., 2011). Similar effects occurred following FAZ2 depletion (Zhou et al., 2015), whereas FAZ9 RNAi altered kinetoplast positioning despite the flagellum remaining attached to the cell body (Sunter et al., 2015a).

Between the FAZ filament and FAZ flagellum domain is the FAZ intracellular domain, which comprises mostly transmembrane proteins that harbour a small intracellular domain and a glycosylated extracellular region for protein interaction. The glycoprotein FLA1, which is homologous to *T. cruzi* GP72, is located at the FAZ intracellular region, specifically at the cell body membrane (Nozaki et al., 1996; Sunter and Gull, 2016). LaCount et al. (2002) demonstrated the importance of FLA1, as in its absence cells did not undergo proper cytokinesis and became multinucleated. In addition, Sun et al. (2013) identified the flagellar membrane protein FLA1-binding protein (FLA1BP), which is essential for flagellum biogenesis and FAZ elongation and interacts with FLA1 in a zipper-like manner to bring about flagellum attachment. These observations of FAZ protein functions highlight that even though proteins might be located in the same domain, their specific sub-localization within the FAZ might reflect distinct functions. Whether this holds true for components of the microtubule quartet domains (yet to be identified) remains to be seen.

The presence of high molecular mass proteins (HMMPs) in the cytoskeleton of several trypanosomatids has previously been reported, although some of their biological functions are still unknown (Ruiz-Moreno et al., 1995; Baqui et al., 1996, 2000a,b). In the non-pathogenic trypanosomatid *Phytomonas serpens*, a megadalton protein named Ps3500 (Baqui et al., 1996, 2000b) presents the same electrophoretic mobility as the muscle giant protein titin with an apparent molecular mass of 3800 kDa (Wang et al., 1979). Ps3500 localizes to the anterior end of the flagellar pocket connecting the cell body cytoskeleton to the flagellum and seems to be an autophosphorylating serine and threonine protein kinase (Baqui et al., 1996, 2000b). In *T. cruzi*, a family of giant proteins (ranging from 700–2500 kDa) located specifically at the FAZ is present in all life cycle stages (Ruiz-Moreno et al., 1995). Additionally, *T. brucei* FLAM3 and ClpGM6 are predicted to have molecular masses of 468 kDa and 660 kDa, respectively (Müller et al., 1992; Rotureau et al., 2014).

Here, we focused our studies on the characterization of the FAZ10 protein in *T. brucei*, which we detected during the course of mass spectrometry analysis to identify novel cytoskeletal HMMPs in kinetoplastids. Here, we characterize FAZ10 as a giant protein of ∼2000 kDa and reveal its precise localization at the FAZ intracellular domain with a FAZ10-specific antibody. Upon depletion of FAZ10, the attachment of the new flagellum is compromised, positioning of kinetoplasts and nuclei is affected and cytokinesis is disrupted. Although FAZ10 is not essential for flagellum and FAZ formation, its depletion affects the orientation of, and distance between, the PFR–axoneme and the FAZ filament. We show that FAZ10 is also required for correct orientation of the cleavage furrow, reinforcing the importance of cytoskeleton proteins in cell division.

## RESULTS

### *Trypanosoma brucei* FAZ10 is a large and repetitive cytoskeletal protein

A mass spectrometry analysis of *T. brucei* HMMPs (>500 kDa) excised from an SDS-PAGE gel was performed and the resulting peptide data was matched against the TriTrypDB (Aslett et al., 2010). We detected Tb927.7.3330, a previously identified FAZ protein (Morriswood et al., 2013), recently named FAZ10 (Sunter et al., 2015a). However, besides its presence in the FAZ, nothing was known about the molecular and cellular biology of this protein.

**Fig. 1.**
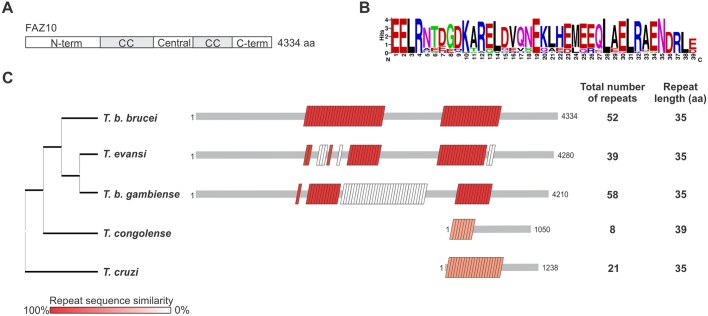
**FAZ10 is a giant repeat-containing protein.** (A) Domain structure of FAZ10 showing N- and C-terminal regions (N-term and C-term, respectively), two coiled-coil (CC) domains and the central region that separates them. (B) Representation of the consensus amino acid sequence for the repeat pattern found in FAZ10 and orthologues. (C) Comparison of the single FAZ10 orthologue present in various trypanosomes. Each box represents a single repeat sequence, which is coloured according to its similarity to the consensus sequence in B.

FAZ10 is encoded by a 13,005 bp long ORF on chromosome 7 and is predicted to be a HMMP (≥502 kDa) based on its sequence in TriTrypDB (http://tritrypdb.org/tritrypdb/). FAZ10 protein structure is characterized by an N-terminal region with no recognizable motifs, two coiled-coil domains (as predicted by COILS; Lupas et al., 1991) separated by a central region, and a C-terminal portion (Fig. 1A). No transmembrane helices were detected using the TMHMM prediction method (Käll et al., 2004). Distributed within the two coiled coil domains of Tb927.7.3330 are 52 nearly identical repeats of 35 amino acids (Fig. 1B,C). Orthologues were detected in other *Trypanosoma* species, each exhibiting similar repeat-containing patterns although with variations in length and number of repeats and diverging up to 57% (*T. cruzi* and *T. con*g*olense*) in amino acid sequence (Fig. 1B,C). Additionally, *T. b. gambiense* and *T. evansi* orthologues also showed another repeated motif, but with a completely different sequence from that observed for FAZ10 (Fig. 1B,C).

### FAZ10 is a giant FAZ protein highly associated with the flagellum

**Fig. 2.**
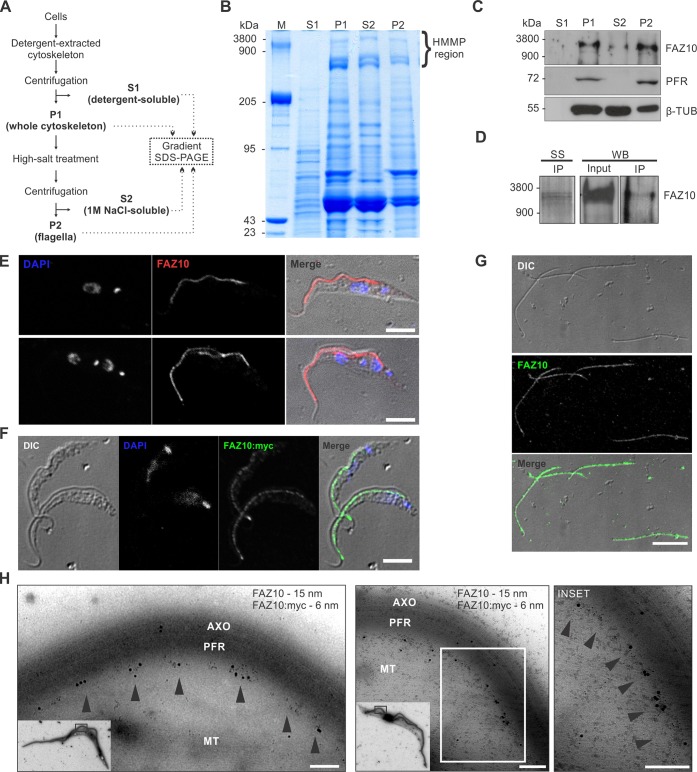
**FAZ10 localizes to the FAZ and is associated with the flagellar cytoskeletal fraction of PCF *T. brucei.*** (A) Flow chart illustrating the preparation of different fractions of the cytoskeleton*.* (B) Linear gradient (2.5–12.5%) SDS-PAGE analysis of cytoskeletal fractions (2×10^7^ cell equivalents/lane). Gels were stained with Coomassie Blue. M, rabbit muscle myofibrils molecular mass standard; S1, detergent-soluble proteins; P1, whole cytoskeleton; S2, cell body proteins soluble in 1 M NaCl; P2, flagella. HMMPs (500–3800 kDa) are indicated with a bracket. (C) Western blot analysis of cytoskeletal fractions (described in B) with anti-FAZ10 antibody. Anti-PFR and anti-β-tubulin antibodies were used to validate the integrity of the cytoskeleton fractions. (D) Immunoprecipitation (IP) of the cytoskeletal fraction with anti-FAZ10 antibody analysed by SDS-PAGE and silver-stained (SS) or by western blotting (WB) with anti-FAZ10 antibody (5×10^8^ cells/lane). (E) Confocal microscopy immunolocalization of FAZ10 protein using anti-FAZ10 antibody (FAZ10, red) in whole cytoskeletons. Nuclei and kinetoplasts were stained with DAPI (blue). (F) Confocal microscopy of cytoskeletons of FAZ10 RNAi+FAZ10::Myc cell line labelled with anti-Myc (FAZ10:myc, green). DAPI (blue) stained nuclei and kinetoplasts. (G) Localization of FAZ10 in intact flagella. DIC and anti-FAZ10 (FAZ10, green) panels are indicated). (H) Immunogold-EM labelling of FAZ10 and FAZ10::Myc. Cytoskeletons were labelled with mouse anti-FAZ10, rabbit anti-Myc antibodies and appropriate secondary antibodies conjugated to colloidal gold (FAZ10–15 nm; Myc–6 nm). Arrowheads, punctate repetitive pattern distribution at the FAZ. Inset is of corresponding enlarged area. AXO, axoneme; PFR, paraflagellar rod; MT, subpellicular microtubules. Scale bars: 5 µm in E,F,G; 200 nm in H.

**Fig. 3.**
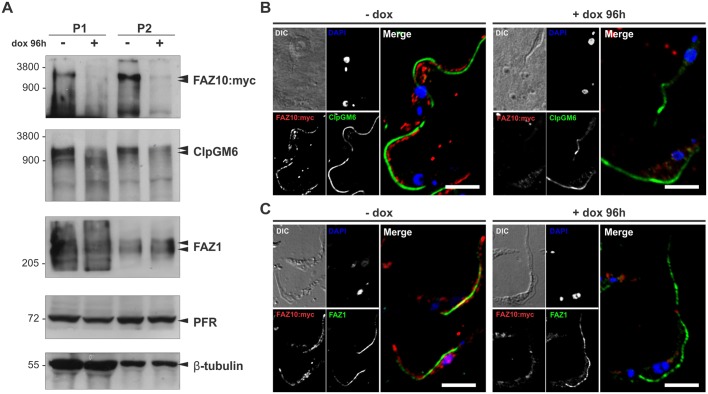
**FAZ10 localizes to the intracellular domain of the FAZ and its depletion modulates ClpGM6 levels.** (A) Western blot analysis (5×10^7^ cell equivalents/lane) of whole-cytoskeleton (P1) or isolated flagella (P2) fractions of uninduced (−dox) or doxycycline-induced (+dox) FAZ10 RNAi+FAZ10::Myc PCF with anti-Myc, anti-ClpGM6, L3B2 (FAZ1), anti-PFR and anti-β-tubulin antibodies. (B,C) Confocal microscopy of FAZ10::Myc cytoskeletons labelled with anti-Myc (FAZ10:myc; red) and anti-ClpGM6 (ClpGM6; green) or L3B2 (FAZ1; green) antibodies before (−dox) and after (+dox; 96 h) doxycycline induction. Nuclei and kinetoplasts were stained with DAPI (blue). Scale bars: 10 µm.

**Fig. 4.**
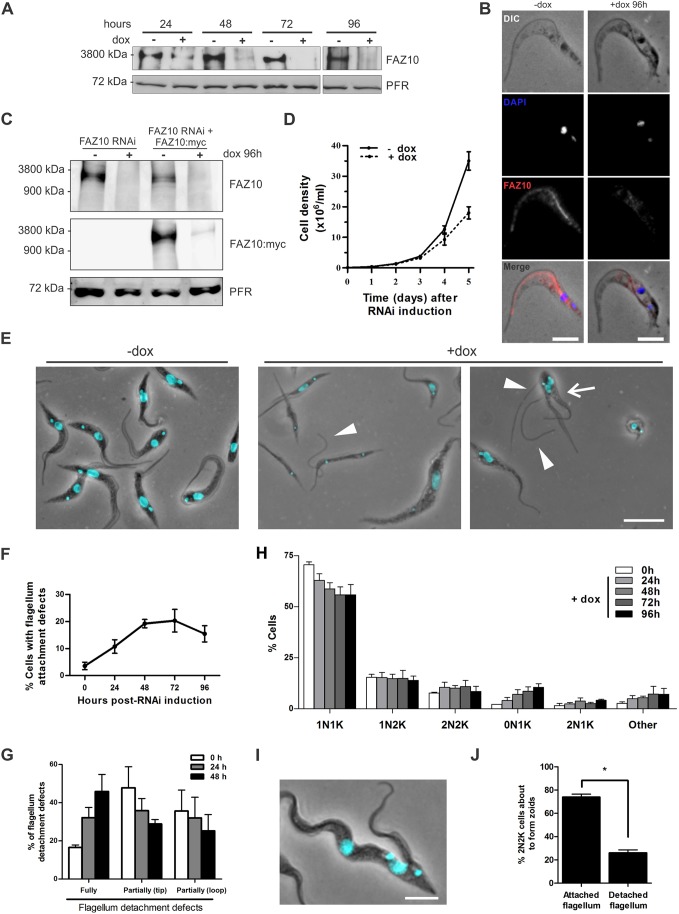
**FAZ10 knockdown in PCF *T. brucei* affects cell growth, morphogenesis, flagellum attachment and furrow positioning.** (A) Western blotting of detergent-extracted cytoskeletons (5×10^6^ cell equivalents/lane) of uninduced (−dox) and doxycycline-induced (+dox) FAZ10 RNAi cells at the timepoints indicated. Anti-FAZ10-labelled FAZ10, with anti-PFR used as a loading control. (B) Immunofluorescence of whole cytoskeletons from uninduced (−dox) and doxycycline-induced (+dox; 96 h) FAZ10 RNAi cells. DIC, DAPI (blue), anti-FAZ10 (FAZ10, red) images are shown. (C) Western blotting of whole cell lysates of uninduced (−dox) and doxycycline-induced (+dox; 96 h) FAZ10 RNAi or FAZ10 RNAi+FAZ10::Myc cells. Anti-FAZ10 and anti-Myc antibodies were used to detect either both FAZ10 and FAZ10::Myc or just FAZ10::Myc. Anti-PFR was used as a loading control. (D) Growth curve of FAZ10 RNAi cells in the presence (+dox) or absence (−dox) of doxycycline. Parasites were induced at 1×10^6^ cells ml^−1^ and counted daily. (E) Fluorescence microscopy of DAPI-stained uninduced (−dox) or induced (+dox; 96 h) FAZ10 RNAi cells. Arrowheads, fully or partially detached flagella. Arrow, cell with abnormal furrow ingression. (F) Quantification of FAZ10 RNAi cells with flagellum attachment defects over time following RNAi induction. *n*>500cells/timepoint. (G) Proportion of cells with flagellum detachment defects at various timepoints after induction with doxycycline. *n*>150 cells/experiment. (H) Quantification of nuclei (N) and kinetoplasts (K) in DAPI-stained FAZ10 RNAi cells over time post-RNAi induction (*n*>250cells/timepoint). (I) DAPI-stained PCF *T. brucei* 2N2K-cell with attached flagella dividing unequally to form 2N1K and zoid cells. (J) Proportion of dividing 2N2K cells about to form zoids with attached or detached flagella after induction of FAZ10 RNAi with doxycycline; *n*>25 cells/experiment; *P<0.05 by *t*-test. Error bars show standard deviation (s.d.) of three experiments. Scale bars: 5 µm in B,I; 10 µm in E.

**Fig. 5.**
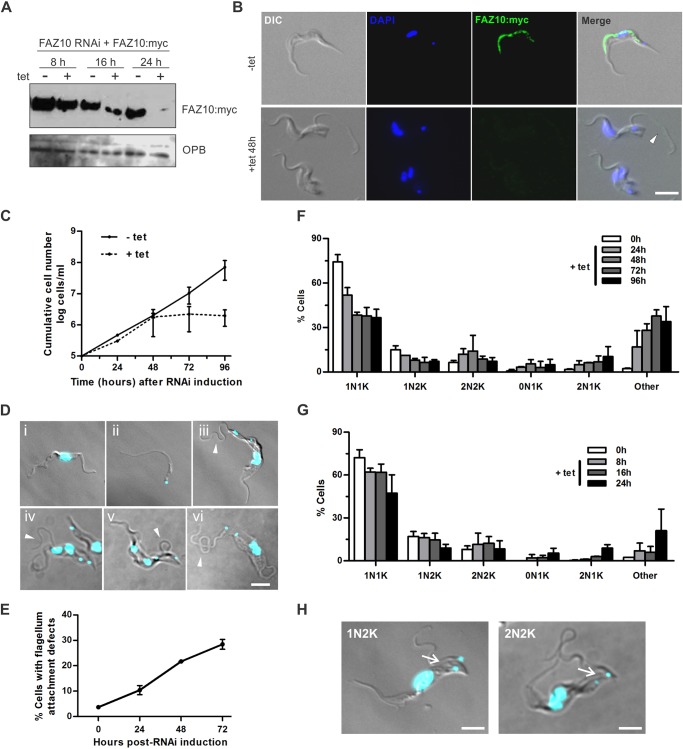
**FAZ10 knockdown in BSF *T. brucei* impairs cell growth and causes flagellum detachment and cytokinesis defects.** (A) Western blotting of 1×10^6^ cells/lane with anti-Myc antibody of whole cell lysates of uninduced (−tet) and tetracycline-induced (+tet) FAZ10::Myc or FAZ10 RNAi+FAZ10::Myc cells. Anti-OPB antibody was used as a loading control. (B) Immunofluorescence of uninduced (−tet) and induced (+tet 48 h) FAZ10 RNAi+FAZ10::Myc cells. DIC, DAPI (blue), anti-Myc (FAZ10:myc, green) images are shown, as indicated. Arrowhead, detached flagellum. (C) Cumulative growth curve of FAZ10 RNAi cells, seeded at 1×10^5^ cells ml^−1^ in the presence (+tet) or absence (−tet) of tetracycline. (D) Fluorescence microscopy of DAPI-stained nuclei and kinetoplasts of FAZ10 RNAi cells at different timepoints (images i–iii and vi are 24 h; images iv and v are 16 h) after induction with tetracycline. Arrowheads, partially or fully detached flagella. (E) Quantification of FAZ10 RNAi cells with flagellum detachment defects over time following RNAi induction. *n*>500 cells/timepoint. (F,G) Quantification of nuclei and kinetoplasts in DAPI-stained FAZ10 RNAi over time post-RNAi induction (*n*>150 cells/timepoint). (H) Fluorescence microscopy of a DAPI-stained FAZ10 RNAi 1N2K and 2N2K cells undergoing unequal cytokinesis 8 h after tetracycline induction. Arrows, cleavage furrows. Error bars show s.d. of three independent experiments. Scale bars: 5 µm.

To facilitate FAZ10 characterization, a mouse polyclonal antibody (anti-FAZ10) was raised against its C-terminal end (amino acids 4085–4334). The serum was used to detect the FAZ10 protein in cytoskeletal preparations by western blotting and to evaluate FAZ10 cellular distribution by immunofluorescence. As FAZ10 is predicted to be a HMMP, cytoskeletal fractions were separated by linear gradient SDS-PAGE (Fig. 2A,B), as has been used extensively to study other megadalton proteins of trypanosomatids (Ruiz-Moreno et al., 1995; Baqui et al., 1996, 2000a,b) and mammals (Wang, 1982). Several HMMP bands were observed in all fractions of the cytoskeleton preparation (Fig. 2B), as compared with titin (3800 kDa) and nebulin (900 kDa) giant proteins from the skeletal muscle myofibrils protein marker (lane M). Anti-FAZ10 western blotting revealed that FAZ10 is a high molecular mass doublet of bands with an apparent mass of 2000 kDa predominantly found in the cytoskeletal (P1) and flagella (P2) fractions (Fig. 2C). Also, the cytoskeletal extract was immunoprecipitated with anti-FAZ10 antibody and western blotting specifically recognized a 2000 kDa cytoskeletal giant protein (Fig. 2D). This is much higher than FAZ10's predicted size of 502 kDa, but use of this antibody in western blotting (see below, Fig. 4A,C) and immunofluorescence analysis of FAZ10 RNAi cells confirmed its specificity (Fig. 4B). C-terminally Myc-tagged FAZ10 (FAZ10::Myc) was detected at the same molecular mass (Fig. 3A, Fig. 4C, Fig. 5A), suggesting that FAZ10 contains more repeat regions than annotated and/or is extensively post-translationally modified. Confocal analysis of whole cells or cytoskeletons revealed that FAZ10 is putatively distributed along the FAZ structure, from the flagellar pocket to the anterior end of the cell (Fig. 2E), where a bulbous enrichment of FAZ10 staining was observed (Fig. 2F; Fig. S1), similar to what has been observed previously by Morriswood et al. (2013) in procyclic form (PCF) *T. brucei* with Ty1-tagged FAZ10. A faint signal was also detected with anti-FAZ10 at the flagellum (Figs S1, S2). Additionally, anti-FAZ10 revealed the presence of FAZ10 along the length of isolated flagella (Fig. 2G; Fig. S2) in agreement with the detection of the protein in the flagellar fraction by western blotting (Fig. 2C). Further, FAZ10::Myc expressed from its endogenous locus in PCF or bloodstream form (BSF) trypanosomes exhibited the same putative FAZ localization as detected by immunofluorescence, although FAZ10::Myc staining was not present on the flagellum itself (Fig. 2F, Fig. 5B; Fig. S1), suggesting perhaps that the Myc tag addition either excludes FAZ10 from the flagellum, or that the Myc tag is masked here by, for example, interaction of FAZ10 with another protein. However, it cannot be ruled out at present that the weak anti-FAZ10 flagellum signal results from a cross reaction of the anti-FAZ10 antibody with a flagellum protein when used in immunofluorescence. A closer examination of FAZ10 in cytoskeletons of PCF *T. brucei* expressing FAZ10::Myc was performed by immunogold-EM double labelling with anti-FAZ10 and anti-Myc antibodies. These antibodies revealed the same punctate pattern distribution along the FAZ, probably corresponding to the ∼122 nm apart junctional complexes (intermembrane staples) that connect the cell body to the flagellum (Höög et al., 2012) (Fig. 2H). Thus, FAZ10 is a giant cytoskeletal protein highly associated with the flagellum located at the intracellular domain of the FAZ.

**Fig. 6.**
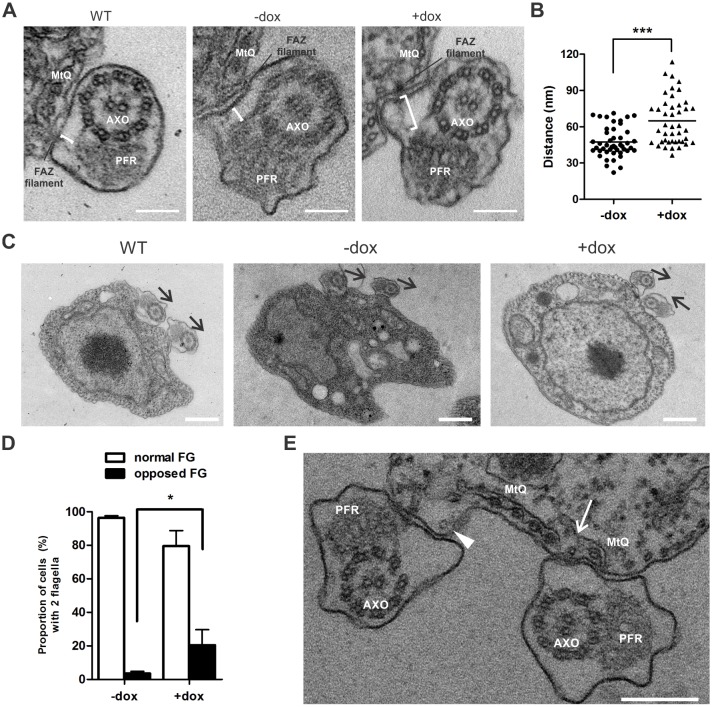
**FAZ10 depletion affects the FAZ filament–PFR distance and the overall organization of FAZ and flagellum structure in PCF *T. brucei*.** (A) Transmission electron microscopy (TEM) of transverse flagellar sections of wild-type (WT), uninduced (−dox) and doxycycline-induced (+dox; 96 h) FAZ10 RNAi PCF *T. brucei*. Brackets mark the distance between the FAZ filament and the distal domain of the PFR. (B) Scatter plot of the distance (nm) between the FAZ filament and the distal domain of PFR for the cell lines in A. The graph represents one of three independent experiments (*n*>40 cells/treatment). (C) TEM images showing the arrangement of duplicated flagella in wild-type (WT), uninduced (−dox) and doxycycline-induced (+dox; 96 h) FAZ10 RNAi PCF cells. Arrows indicate the orientation of the PFR-axoneme. (D) Proportion of cells with normal or opposed flagella (FG) as seen in C (*n*>100 cells/experiment). (E) TEM image of 96 h doxycycline-induced FAZ10 RNAi PCF cell showing opposed flagella, disorganization of the duplicated microtubule quartet (MtQ) (arrow) and disruption of the subpellicular microtubules (arrowhead). AXO, axoneme; PFR, paraflagellar rod. *P<0.05, ***P<0.0001 by *t*-test. Error bars show s.d. of three independent experiments. Scale bars: 100 nm in A,C; 500 nm in E.

**Fig. 7.**
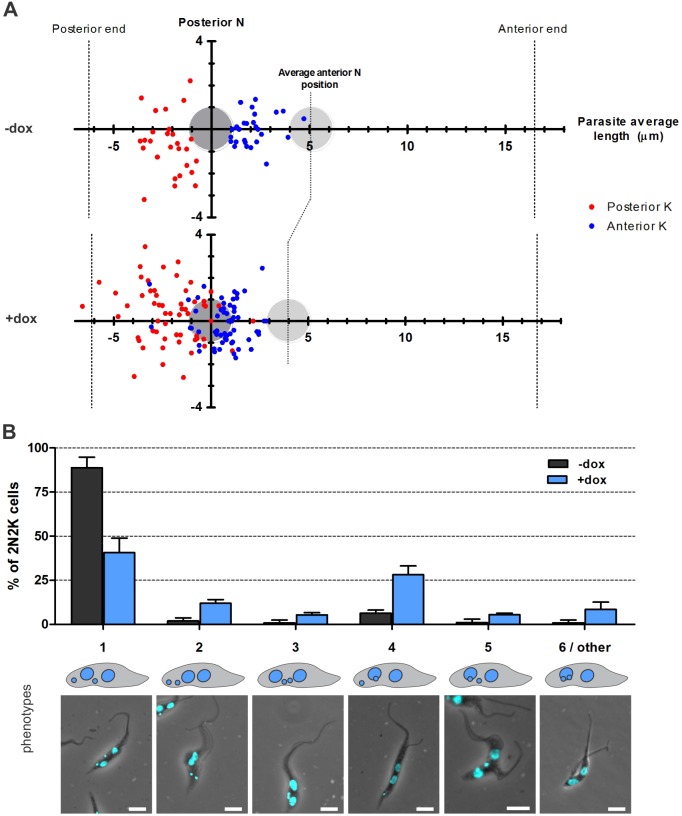
**FAZ10 is essential for segregation and positioning of the nuclei and kinetoplasts in PCF *T. brucei*.** (A) Schematic illustration of the average position of the anterior nucleus (light grey circle) and the positions of the anterior (red dots) and posterior (blue dots) kinetoplasts relative to the posterior nucleus (dark grey circle, *y* axis) in 2N2K cells (pre-abscission) in uninduced (−dox) and doxycycline-induced (+dox) FAZ10 RNAi PCF cells from various timepoints after RNAi induction (*n*>90 cells/experiment, graph represents one of three independent experiments). The positions of the nuclei and kinetoplasts were measured as described in Materials and Methods. N, nucleus; K, kinetoplast. (B) Proportions of 2N2K cells (from various timepoints) exhibiting the arrangements of nuclei and kinetoplasts showed in A, as visualized by DAPI staining. Error bars show s.d. of three independent experiments. Scale bars: 5 µm.

**Fig. 8.**
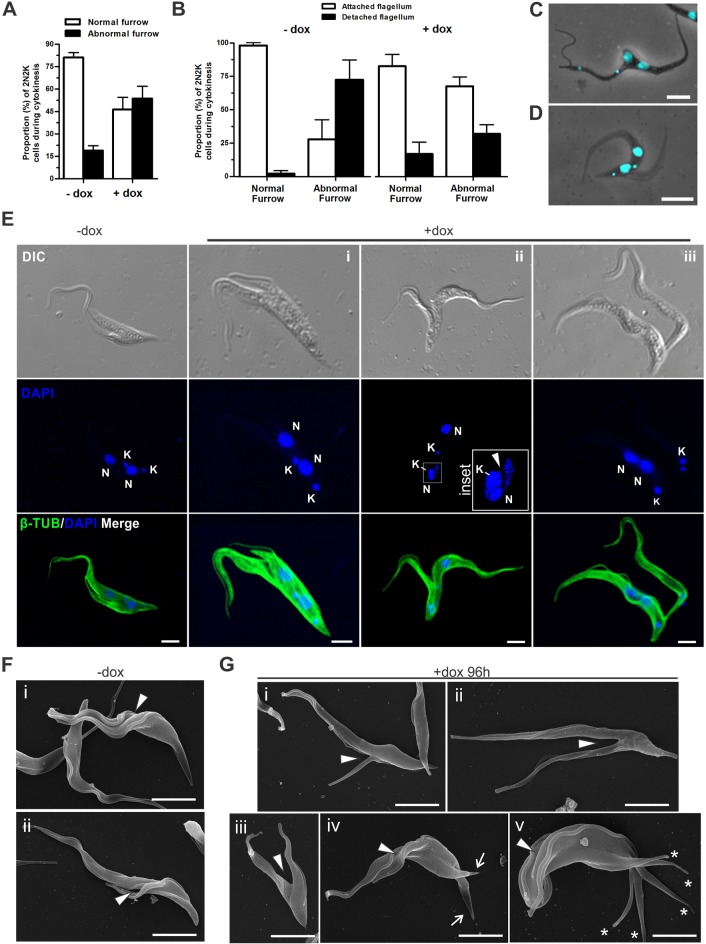
**FAZ10 is required for correct cleavage furrow placement in PCF *T. brucei*.** (A) Percentage of 2N2K cells with normal or abnormal placement of the cleavage furrow in uninduced (−dox) or doxycycline-induced (+dox) FAZ10 RNAi cells at various timepoints after induction (*n*>65 cells/experiment). (B) Percentage of 2N2K cells with flagellum attachment defects correlated with whether they exhibit normal or abnormal furrow placement, in uninduced (−dox) or doxycycline-induced (+dox; 96 h) FAZ10 RNAi PCF cells at various timepoints after induction (*n*=65 cells/experiment). (C,D) Merged DAPI-stained and DIC images of doxycycline-induced FAZ10 RNAi cells displaying aberrantly positioned cleavage furrows with (C) incorrect nucleus and kinetoplast placement and detached flagellum, or (D) correct nucleus and kinetoplast placement and attached flagella. (E) Confocal microscopy of whole cytoskeletons from uninduced (−dox) and doxycycline-induced (+dox; 96 h) FAZ10 RNAi cells. Cells were labelled with anti-β-tubulin antibody (β-TUB) and nuclei (N) and kinetoplasts (K) were stained with DAPI. Merged images are shown as indicated. Inset/arrowhead (ii) indicates the ingression of the furrow through the nucleus. (F,G) Scanning electron micrograph (SEM) images of uninduced (−dox) and doxycycline-induced (+dox, 96 h) FAZ10 RNAi cells. Arrowheads, cleavage fold generation or cleavage furrow ingression. Asterisks, spicules. Arrows, defined posterior ends of forming daughter cells. Error bars show s.d. of three independent experiments. Scale bars: 5 µm.

To further investigate FAZ10 size and distribution related to other large FAZ proteins such as ClpGM6 and FAZ1, a PCF *T. brucei* FAZ10 RNAi cell line expressing FAZ10::Myc from the endogenous locus was generated (see Figs 4, 6–8 for its full characterization). Western blotting analysis was performed to detect FAZ10, ClpGM6 and FAZ1 in the whole cytoskeleton (P1) and flagella (P2) fractions in control or doxycycline-induced FAZ10 RNAi cells. Similar to what has been observed for FAZ10, ClpGM6 is also a giant protein with a molecular mass of ∼1500 kDa (Fig. 3A), based on its electrophoretic mobility when compared with the protein marker. This provides a more precise molecular mass for ClpGM6 than previous studies (Müller et al., 1992; Hayes et al., 2014), where ClpGM6 was detected as several protein bands >250 kDa in size. Additionally, the lack of FAZ10 seems to cause a noticeable reduction in ClpGM6 (FAZ flagellum domain) protein levels (Fig. 3A). In contrast, knockdown of FAZ10 did not alter cellular levels of FAZ1 (Fig. 3A), a cell-body-side FAZ protein (FAZ filament domain) predicted to be 210 kDa in molecular mass (Kohl et al., 1999) that is mostly solubilized when flagella are isolated from the cytoskeleton (Rotureau et al., 2014).

Next, cytoskeletons were co-labelled with anti-FAZ10 and with either anti-ClpGM6 or the antibody L3B2 to visualize FAZ1, before and after induction of FAZ10 RNAi. Confocal analysis revealed that FAZ10 does not seem to colocalize with ClpGM6 or to completely colocalize with FAZ1 (Fig. 3B,C). Further, FAZ10 silencing does not affect ClpGM6 or FAZ1 distribution in the cell (Fig. 3B,C). Hence, we propose that FAZ10, given its apparent lack of transmembrane domains, its association with isolated flagella, periodic localization along the FAZ and close proximity to FAZ1 and ClpGM6, localizes to the FAZ intracellular domain (intermembrane staples) (Höög et al., 2012; Sunter and Gull, 2016).

### Knockdown of FAZ10 affects cell proliferation, flagellum attachment and inhibits segregation of nuclei and kinetoplasts in *T. brucei*

The function of FAZ10 was investigated using RNAi, initially in PCF *T. brucei*. Upon 24 h of RNAi induction, FAZ10 protein levels decreased substantially and were barely detectable by western blotting or immunofluorescence by 72–96 h post-induction (Fig. 4A,B). FAZ10 depletion was also confirmed by introducing FAZ10::Myc into the FAZ10 RNAi cell line and western blotting with anti-Myc antibody (Fig. 4C). Depletion of FAZ10 was accompanied by a decrease in growth rate from 3 days post-induction, indicating that FAZ10 is necessary for normal cell proliferation (Fig. 4D). Furthermore, FAZ10 depletion caused morphological defects in cell shape and size (Fig. 4E). In addition, induced cells exhibited flagellum attachment abnormalities (Fig. 4E,F), with the proportion of cells with one or more flagella fully detached from the cell body increasing substantially by 48 h post-RNAi induction (Fig. 4G). As flagellar detachment can result in defective nuclei and kinetoplast segregation and cytokinesis defects, cell cycle progression in DAPI-stained FAZ10 RNAi cells was examined. Cells containing abnormal numbers of nuclei (N) and kinetoplasts (K) were observed to accumulate over time (Fig. 4H), including zoids (Robinson et al., 1995) [containing a single kinetoplast (0N1K)] and 2N1K cells, which comprised ∼11% and 5% of the population, respectively, by 96 h post-induction. The origin of these cells seemed to be 2N2K cells that were unable to divide in the right plane, generating unequal daughter cells (Fig. 4I). The lower numbers of 2N1K cells compared with zoids can be explained by 2N1K cells continuing to replicate their organelles and becoming multinucleated cells (Fig. 4H, ‘Other’ cells). Of the 2N2K cells undergoing unequal cell division, only ∼25% exhibited any obvious flagellar detachment (Fig. 4I,J). Thus, it is possible that depletion of FAZ10 might also influence cleavage furrow positioning, independently of its role in maintaining flagellar attachment.

To investigate FAZ10 function in BSF *T. brucei*, a FAZ10 RNAi cell line endogenously expressing FAZ10::Myc was generated. Induction of FAZ10 RNAi resulted in depletion of FAZ10::Myc protein to undetectable levels after 24–48 h post-induction, as monitored by western blotting and immunofluorescence (Fig. 5A,B). Similar to procyclic cells, FAZ10 localized to the FAZ structure (Fig. 5B). FAZ10 knockdown impaired cell growth from 24 h post-induction (Fig. 5C) and resulted in the accumulation of cells that were aberrant with respect to their size and shape (Fig. 5Di,ii,iii), and flagellum attachment (Fig. 5Div,v,vi). Flagellum attachment defects were detected from 16 h post-induction, initially in small numbers and increased over time following FAZ10 ablation with detached flagella being detected in ∼25% of the cells by 72 h post-induction (Fig. 5E). Although the majority of detached flagella observed were unattached to the cell body along their entire length (Fig. 5Dvi), flagella detached in loops or just at the anterior tip were also observed (Fig. 5Div,v), as in the PCF (Fig. 4G). A small increase in 2N2K cells, many with incompletely segregated nuclei, was observed over the first 48 h post-induction (Fig. 5F–H). Significant numbers of multinucleated cells were also observed from 24 h post-induction (Fig. 5F,G, ‘Other’ cells). Similar to procyclic cells, zoids were also observed, although in smaller numbers. These arose from 1N2K cells that divided prematurely prior to completion of mitosis as well as from aberrant division of 2N2K cells (Fig. 5D,H).

### FAZ10 is responsible for the organization of the FAZ and flagellar structures

To investigate whether the cell cycle and flagellum attachment defects observed could have occurred as a consequence of the malformation of essential structures of the trypanosome flagellum, the axoneme, FAZ and PFR were examined by immunofluorescence of isolated PCF flagella and cytoskeletons using β-tubulin, FAZ1 and PFR antibodies. No alterations in protein distribution were observed (Figs S2, S3), consistent with western blotting data in Fig. 3A, indicating that FAZ10 depletion did not impair the formation of these structures. To investigate further the flagellar and FAZ structure following FAZ10 RNAi, cells were examined by transmission electron microscopy (TEM). Although FAZ10-depleted cells still formed the PFR and FAZ (Figs S2, S3), by TEM an increased distance between the PFR and the intracellular FAZ was observed (mean±s.d.; 60.84 nm±17.51 nm, induced samples vs 47.31 nm±12.35, uninduced samples; Fig. 6A,B), indicating a loss of proper linkage between these structures. Moreover, in ∼20% induced cells with two flagella, the axoneme–PFR structures were no longer orientated in the same direction as in wild­-type or uninduced cells; instead, one flagellum was rotated such that its axoneme faced the axoneme of the second flagellum (Fig. 6C–E). It is possible that this could have occurred as a result of a weakened flagellum attachment following FAZ10 depletion. Besides these effects, the arrangement of subpellicular microtubules underlying the flagellum also showed some disorganization (Fig. 6E, arrowhead).

Thus, although FAZ10 is probably not essential for the formation of the axoneme, PFR and FAZ structure, it likely plays roles in their overall organization, with direct implications for their functionality.

### Lack of FAZ10 compromises nucleus and kinetoplast segregation and positioning

FAZ10-depleted PCF and BSF cells also exhibited mispositioned nuclei and kinetoplasts after mitosis, which led to the accumulation of aberrant cells after cytokinesis (Fig. 4E,H,I; Fig. 5D,F–H). This phenomenon was analysed in more detail in PCF *T. brucei* by tracking the position of the duplicated kinetoplasts relative to the posterior nucleus in 2N2K cells (Fig. 7A). Compared with uninduced cells, posterior kinetoplasts in FAZ10-depleted cells had a broader distribution range, whereas the anterior kinetoplasts were distributed significantly more posteriorly with many overlaying or being located posterior to the posterior nucleus (Fig. 7A). Additionally, the internuclear distance was reduced (Fig. 7A). A number of abnormal rearrangements of nuclei and kinetoplasts were present in 2N2K cells (Fig. 7B), most commonly with the anterior kinetoplast superimposed over the posterior nucleus (Fig. 7B, phenotype 4).

### FAZ10 is essential for defining the cleavage furrow path during cytokinesis

Cytokinesis also seemed to be affected following FAZ10 knockdown. Close examination of FAZ10-depleted cells showed that in 2N2K cells undergoing cytokinesis, the furrow was either incorrectly positioned (asymmetrical; Fig. 4I) or incorrectly timed (occurring prior to completion of nucleus and kinetoplast duplication and segregation; Fig. 5H) in a much greater proportion of dividing cells compared with uninduced cells (Fig. 8A). We hypothesized that flagellum detachment was responsible for that defect. However, whereas most dividing cells with detached flagella in the uninduced population exhibited defective furrow ingression (Fig. 8B,C), defective furrow ingression in cells lacking FAZ10 was mostly not associated with any obvious defective flagellum attachment, as 65–70% of aberrantly dividing cells did not display detached flagella (Fig. 8B,D). This is in agreement with the percentage of unequally dividing cells forming zoids with attached flagella (Fig. 4I,J). These observations were confirmed by visualizing the highly asymmetric segregation of the cell body and the nuclei and kinetoplasts by means of β-tubulin antibody labelling and DAPI staining (Fig. 8E). Importantly though, defective furrow ingression also occurred when nuclei and kinetoplasts were correctly positioned (Fig. 4J, Fig. 8Ei), arguing that FAZ10 depletion could result in defective furrow ingression in the absence of obvious flagellar attachment or nucleus and/or kinetoplast positioning defects.

Similar observations were made by scanning electron microscopy (SEM). Cleavage fold generation and cleavage furrow ingression (from the anterior to the posterior) were observed to progress symmetrically in the majority of dividing cells in uninduced populations (Fig. 8F). However, in induced populations, these processes were observed to occur asymmetrically (Fig. 8G). It was also noticeable that the membranes of induced cells displayed reduced undulation compared with uninduced cells, supporting a role for FAZ10 in maintaining cell morphology. A recent detailed SEM study of trypanosome cytokinesis showed that distinct daughter cell posterior ends are visible (but not separated) after cleavage fold formation but prior to cleavage furrow ingression (Wheeler et al., 2013). However, this order of events was disrupted in some FAZ10-depleted cells. Cells with visible cleavage furrows but lacking definition of daughter cell posterior ends were observed (Fig. 8Giii). Additionally, in FAZ10-depleted cells, SEM also revealed dividing cells with two flagella and a cleavage fold, but no anterior to posterior cleavage furrow that had remodelled their posterior end into two separated tips or several spicules (Fig. 8Giv,v).

Our observations indicate that FAZ10 might play a role in the definition of the path for cleavage furrow ingression during the early steps of cytokinesis and might inhibit proper cytokinesis through several mechanisms: flagellar detachment, reduced segregation and mispositioning of the duplicated nuclei and kinetoplasts, and aberrant placement and possibly timing of the cleavage furrow.

## DISCUSSION

We report here the functional characterization of *T. brucei* FAZ10, apparently the largest protein investigated and characterized in *T. brucei* to date. Although the annotated *FAZ10* in TriTrypDB is predicted to encode a 502 kDa protein, FAZ10 was detected here by western blot analysis as a doublet of proteins of ∼2000 kDa, about four times larger than expected. As there is an abundance of repetitive elements in FAZ10, it is possible that these regions might not have been correctly assembled during sequencing and are actually more extensive than annotated in TriTrypDB. Furthermore, we showed here for the first time, using linear gradient SDS-PAGE and HMMP standards that the ClpGM6 protein is higher than its predicted 660 kDa size, as observed for FAZ10. Several studies have described HMMPs in trypanosomatids (Ruiz-Moreno et al., 1995; Baqui et al., 1996, 2000b), in other protozoa (Mattei et al., 1992; Garcia and Abrahamson, 1995) and although the roles of most of the HMMPs remain unclear, several of them have been localized to the cytoskeleton and in particular to the FAZ. Interestingly, where their functions have been determined, most HMMPs seem to play important roles in cell morphogenesis (Hayes et al., 2014; Rotureau et al., 2014).

Trypanosomatid giant cytoskeletal proteins have also been detected as doublets in *Phytomonas*, *Leptomonas* and *Leishmania* (Baqui et al., 2000b), as reported for the elastic titin (Wang et al., 1979; Labeit and Kolmerer, 1995). A similar migration pattern was observed for FAZ1, FLAM3 and ClpGM6 (Vaughan et al., 2008; Hayes et al., 2014; Rotureau et al., 2014), and our unpublished observations suggest that this behaviour might be a common feature of HMMPs. Although FAZ10 protein degradation might have occurred during the isolation of cytoskeletons and flagella, especially if it is particularly susceptible to proteolysis as was previously suggested for titin (Wang et al., 1979), several protease and phosphatase inhibitors were added to minimize these effects. It is therefore possible that the FAZ10 doublet could represent allelic variation resulting from heterogeneity in the number of repeats, or that the two bands could represent different post-translational modifications, as has been proposed for the FAZ1 doublet (Vaughan et al., 2008).

Additionally, FAZ10 partially colocalizes with FAZ1 and is localized close to ClpGM6, although these proteins do not overlap at any point. As FAZ10 co-purifies with isolated flagella but does not contain a transmembrane domain, we suggest that FAZ10 might be localized to the FAZ intracellular domain, most likely to the intermembrane staples (Sunter and Gull, 2016), as indicated by immunogold EM of *T. brucei* cytoskeletons. This is, to our knowledge, the first protein to be localized to the intermembrane staples in any kinetoplastid. It is worth highlighting here that our immunogold EM data indicate that FAZ10 does not form a linear filament as suggested by lower resolution immunofluorescence microscopy, but instead has a repeating periodic localization along the length of the FAZ. FAZ10 localization to the FAZ intracellular domain is consistent with other FAZ proteins that display flagellum attachment and/or nucleus or kinetoplast positioning defects following RNAi depletion. Depletion of FAZ10 resulted in an increased distance between the distal domain of the PFR and the junctional complexes of the FAZ intracellular domain, indicating that FAZ10 might act to tether these structures together, providing the first mechanistic insights as to why FAZ10 is important for flagellar attachment. Interestingly, in *Leishmania*, an eYFP–FAZ10 fusion protein was recently shown to localize as a horseshoe or ring around the flagellum at the point it exits the flagellar pocket in promastigotes and to extensive attachments present around the constriction of the flagellar pocket neck in amastigotes (Wheeler et al., 2016). Although there are key differences in FAZ structure between the two organisms (Wheeler et al., 2016), it seems likely that FAZ10's role in flagellum attachment will be conserved in *Leishmania*.

Our data suggest that FAZ10 is essential for several biological functions. Firstly, FAZ10 is required for flagellum attachment as its knockdown led to ∼30% of cells displaying detached flagella in both PCF and BSF parasites, similar to the proportion of flagellum attachment defects that occurred following FAZ1 depletion (Vaughan et al., 2008). Defects in flagellum orientation with respect to the cell body were also observed. Several other FAZ proteins are known to participate in flagellum attachment (LaCount et al., 2002; Vaughan et al., 2008; Sun et al., 2013; Rotureau et al., 2014), but it seems that these cannot entirely compensate for the absence of FAZ10.

In addition to flagellum attachment, FAZ10 plays important roles in cell division. Following FAZ10 depletion, cells accumulated with abnormal numbers of nuclei and kinetoplasts that were often mispositioned or not fully segregated, and it is likely that these effects, at least in part, can be attributed to defective flagellar attachment. The flagellum and FAZ have been shown to be crucially important for the repositioning of organelles after their duplication (Zhou et al., 2011; Sun et al., 2013; Hayes et al., 2014) and it has been reported that formation and elongation of the new flagellum and FAZ provide the force necessary for the movement of the new basal body and kinetoplast towards the posterior end of the cell (Absalon et al., 2007). We hypothesize that the newly formed flagellum and FAZ, which exhibit disorganization and decreased attachment to the cell body, have reduced traction with which to bring about the movement of these organelles following their duplication, thus resulting in a range of aberrant nucleus and kinetoplast positions in 2N2K cells. However, a direct role in organelle positioning independently of flagellar detachment cannot be entirely ruled out at this stage, as in many 2N2K cells exhibiting aberrant nucleus and kinetoplast positioning, the flagella were not obviously detached. Indeed, depletion of cytoskeletal-associated protein AIR9 (May et al., 2012) or phosphatase PP1-3 (Gallet et al., 2013) resulted in nucleus and/or kinetoplast positioning defects in the absence of flagellum attachment defects.

Defective flagellar attachment is also known to disrupt or inhibit cytokinesis. For instance, FAZ1 knockdown cells, which are able to form a FAZ, albeit an aberrant one, display a range of detachment defects of the flagellum, leading to aberrant nucleus segregation and defects in cytokinesis (Vaughan et al., 2008). Similarly, knockdown of FLA1 leads to flagellum detachment, resulting in cytokinesis arrest and the formation of multinucleate cells (LaCount et al., 2002). It should be noted that flagellar detachment does not always lead to defects in cytokinesis. RNAi silencing of the FLA1-binding protein, FLA1BP, causes flagellum detachment but does not affect cell division (Sun et al., 2013). Here, although FAZ10-depleted cells with detached flagella mostly underwent aberrant cytokinesis, characterized by asymmetrical ingression of the cleavage furrow, the majority of 2N2K cells displaying defective cytokinesis (∼75%) had attached flagella, arguing that FAZ10 also plays a direct role in cytokinesis.

Additionally, some FAZ10-depleted PCF cells were observed to remodel their posterior end in the absence of cleavage furrow ingression from the anterior end, which bears similarity to two previously reported phenotypes: remodelling of the daughter cell posterior ends (which usually occurs late in cytokinesis) proceeding in the absence of a cleavage furrow, as observed following KAT60b or KAT60c depletion (Benz et al., 2012), or the ingression of a cleavage furrow from the posterior-to-anterior as has been reported following CIF1 (a putative close neighbour or binding protein of FAZ10) or CIF2 depletion (Zhou et al., 2016a,b). Whatever the molecular mechanism for posterior end remodelling following FAZ10 depletion, our observations indicate that FAZ10 might play a role in the definition of the path for cleavage furrow ingression during the early steps of cytokinesis.

Further, in BSF cells, in addition to 2N2K cells dividing unequally, 1N2K cells with attached flagella were observed to undergo premature and unequal cytokinesis. It is possible that mitosis or separation of the segregated nuclei could be delayed or inhibited in FAZ10-depleted cells, meaning that at the normal time of cytokinesis, the cell still only has one nucleus. It is unlikely that FAZ10 depletion inhibits mitosis in BSF trypanosomes, as that usually prevents cytokinesis (Hammarton et al., 2003). However, many 2N2K cells were observed to have closely positioned nuclei, suggesting that nucleus segregation was compromised following FAZ10 knockdown in the BSF as was also demonstrated in the PCF, and that this could account for the dividing 1N2K cells. However, a second possibility is that depletion of FAZ10 could stimulate premature cytokinesis such that it occurs prior to mitosis in some 1N2K cells. Thus, FAZ10 seems to be required for determining cleavage furrow positioning and possibly for the timing of furrow ingression, independently of its role in flagellar attachment.

Taken together, our data indicate that depletion of FAZ10 results in several different phenotypes likely mediated by FAZ10 depletion destabilizing other FAZ proteins, such as ClpGM6, as demonstrated here. Many studies have shown that FAZ architecture relies on a number of proteins with different functional roles and displaying specific localizations within the FAZ (Absalon et al., 2007; Vaughan et al., 2008; Zhou et al., 2011; Hayes et al., 2014; Rotureau et al., 2014; Hu et al., 2015; Sunter and Gull, 2016). Roles in flagellar attachment and organelle positioning, as defined here for FAZ10, are common to many FAZ proteins. However, FAZ10 is the first FAZ protein to be directly linked to controlling the trajectory and possibly also the timing of the cleavage furrow. Given its giant size and repetitive nature, it is tempting to speculate that FAZ10 might act as a scaffold for regulatory signalling molecules at the site of cytokinesis initiation. Studies to isolate binding partners of FAZ10 are now required to elucidate the molecular mechanisms by which it operates.

## MATERIALS AND METHODS

### Bioinformatics

TriTrypDB (http://tritrypdb.org/tritrypdb/) was used to search for FAZ10 orthologues. Sequences similarities were analysed by BLAST (http://blast.ncbi.nlm.nih.gov/Blast.cgi) and alignment analyses were performed with Clustal Omega (http://www.ebi.ac.uk/Tools/msa/clustalo/). Sequence logos were generated using WebLogo version 2.8.2 (http://weblogo.berkeley.edu/logo.cgi).

### Trypanosomes and cell culture

All cell lines were derivatives of *Trypanosoma brucei* strain 427. For RNAi, the procyclic 29-13 and bloodstream form 90-13 host cell lines were used (Wirtz et al., 1999). PFC were grown in SDM-79 medium (LGC Bio, São Paulo, Brazil) supplemented with hemin and 10% FBS (Life Technologies, CA) at 28°C and 5% CO_2_ (Brun and Schonenberger, 1979). BSF parasites were cultured in HMI-9 medium (Life Technologies) supplemented with 20% FBS at 37°C and 5% CO_2_ (Hirumi and Hirumi, 1989).

### RNAi cell lines and transfection

For the construction of the FAZ10 RNAi cell line, a 575 bp target fragment of the *FAZ10* coding sequence (Tb927.07.3330), selected using RNAit software (http://trypanofan.bioc.cam.ac.uk/software/RNAit.html) (Redmond et al., 2003), was cloned into the tetracycline-inducible p2T7-177 vector (Wickstead et al., 2002). To construct FAZ10::Myc cell lines, the last 900 bp of the *FAZ10* coding sequence without the stop codon were cloned (*Hind*III and *Xba*I) into the pNATxTAG vector (Alsford and Horn, 2008). Transfections were performed using either a Gene Pulser Electroporation System (Bio-Rad; PCF) (McCulloch et al., 2004) or an AMAXA Nucleofector II system (programe X-001; BSF) (Burkard et al., 2007). Transformants were selected and maintained in the presence of G418 (15 µg ml^−1^), hygromycin (50 µg ml^−1^) and zeocin (10 µg ml^−1^) for PCF and G418 (2.5 µg ml^−1^), hygromycin (5 µg ml^−1^) and phleomycin (2.5 µg ml^−1^) for BSF. FAZ10::Myc cell lines were maintained in the presence of blasticidin (10 µg ml^−1^). RNAi was induced by adding 1 µg ml^−1^ doxycycline or tetracycline to cells of starting density 1×10^6^ cells ml^−1^ (PCF) and 1×10^5^ cells ml^−1^ (BSF), refreshing the drug at each culture dilution (Wirtz et al., 1999).

### DAPI staining

Cells were fixed in −20°C methanol for at least 1 h and stained with 4′,6-diamidino-2-phenylindole (DAPI), and nuclei and kinetoplasts per cell were counted using an epifluorescence microscopy DMI4000 Leica [(Ribeirão Preto Medical School, University of São Paulo (FMRP/USP)] or Zeiss Axioskop2 microscope/Axiocam MRM camera [University of Glasgow (UoG)].

### Morphogenetic measurements

Fluorescence microscopy images of DAPI-stained cells were used to evaluate kinetoplast and nucleus positioning. ImageJ (National Institutes of Health, Bethesda, MD; http://imagej.nih.gov/ij/) was used to measure the position of kinetoplasts and nuclei in 2N2K cells. The centre of the posterior nucleus was used as reference for the coordinates of the kinetoplasts and anterior nucleus. A straight line connecting the two nuclei was used to define the longitudinal axis of the cell (posterior–anterior end).

### Polyclonal FAZ10 antibody

The coding sequence for the last 250 amino acids of Tb927.7.3330 (without the stop codon) was fused to the glutathione-S-transferase (GST) coding sequence in the pGEX-4T-1 vector (GE Healthcare) using the *Eco*RI and *Xho*I restriction sites. GST:CtermFAZ10 was expressed and purified from BL21star *Escherichia* *coli* using glutathione–Sepharose according to the manufacturer's instructions. Purified GST:CtermFAZ10 recombinant protein was then emulsified with PBS and Freund's complete adjuvant and injected subcutaneously into female Balb/C mice (6 weeks old). Three additional boosts with incomplete adjuvant were performed at 2 week intervals. Blood was collected from the orbital plexus (Donovan and Brown, 2006) and the serum (which was confirmed by western blot to be unreactive against GST) used directly in subsequent analyses. All experiments with mice were approved in accordance with the Ethical Principles on Animal Experimentation (Protocol no. 014/2012) of the University of São Paulo (COBEA).

### Cytoskeleton and flagella isolation

Whole cytoskeletons and flagella and other cell fractions were obtained as described by Baqui et al. (2000b). Briefly, to obtain cytoskeletons, cells were resuspended in ice-cold lysis buffer (50 mM HEPES pH 6.9; 0.1 mM EDTA; 5 mM EGTA and 2 mM MgCl_2_) containing 1% NP-40 (IGEPAL CA-630) and the following protease and phosphatase inhibitors: 2 µg ml^−1^ aprotinin, 5 µg ml^−1^ antipain, 0.5 mM phenylmethylsulfonyl fluoride, 10 µM leupeptin, 1 mM DTT, 0.5 mM benzamidine, 1 mM sodium orthovanadate and 5 mM sodium fluoride. Flagella were obtained by resuspending the cytoskeletons in 50 mM Tris-HCl pH 7.2, 1 M NaCl plus the inhibitors as described above and incubating on ice for 45 min prior to centrifuging at 10,000 ***g*** for 10 min at 4°C to obtain isolated flagella (pellet) and cell body (supernatant).

### Immunoprecipitation

Briefly, *T. brucei* cytoskeletons (2×10^9^ cells) were resuspended in 1 ml RIPA buffer (50 mM Tris-HCl pH 8.0, 150 mM NaCl, 0.1% SDS, 0.5% sodium deoxycholate, 1% NP-40) in the presence of protease and phosphatase inhibitors. After centrifugation, the resulting supernatant was diluted in an equal volume of T-NET buffer (20 mM Tris-HCl pH 8.0, 100 mM NaCl, 1 mM EDTA and 0.5% NP-40) and incubated with pre-immune mouse serum (1:200) for 2 h at 4°C with agitation. After further centrifugation, the supernatant was removed to a new tube and a suspension of protein A–Sepharose (GE Healthcare) was added and incubated for 30 min at 4°C with agitation. The supernatant was collected in a new tube and incubated with anti-FAZ10 antibody (this study, see ‘Polyclonal FAZ10 antibody’ above; 1:50) overnight at 4°C. Thereafter, the same procedure was repeated by addition of protein A–Sepharose and centrifugation. The immunocomplexes were resuspended in sample buffer for analysis by SDS-PAGE.

### SDS-PAGE and western blotting

SDS-PAGE analysis was performed as described by Baqui et al. (2000b). Samples were analysed using a 2.5–12.5% polyacrylamide gradient gel (PCF; 0.5–2×10^7^ cells/lane) or a NuPAGE 3–8% Tris-acetate gradient gel (BSF; 1×10^6^ cells/lane) and rabbit skeletal muscle myofibrils were used as protein mass standards (Wang, 1982; Baqui et al., 1996). For western blotting analysis, the following primary antibodies were used: anti-FAZ1 (L3B2, 1:2, a kind gift from Keith Gull, University of Oxford, UK; Kohl et al., 1999); anti-PFR (1:2000, a kind gift from Sérgio Schenkman, UNIFESP, Brazil); anti-β-tubulin (1:10,000, Sigma, cat.# T8328); anti-oligopeptidase B (OPB; 1:1000, a kind gift from Jeremy Mottram, University of York, UK; Munday et al., 2011); anti-Myc, clone 9E10 (Evan et al., 1985). Then, membranes were incubated with goat anti-mouse (1:12,000, Pierce, cat.# 31430) or goat anti-sheep (for anti-OPB; 1:10,000, Santa Cruz Biotechnology, cat.# sc-2916) IgG peroxidase-conjugated antibody and the signal detected with enhanced chemiluminescence (GE Healthcare) or West Pico/Dura detection reagent (Pierce), according to the manufacturer's instructions.

### Immunofluorescence

Immunofluorescence was performed as described by Baqui et al. (2000b). We used the following primary antibodies: anti-β-tubulin (1:500; as above), anti-PFR (1:250; as above), anti-FAZ1 L3B2 (1:5; as above), anti-Myc antibody clone 4A6 (1:300, Millipore, cat.#05-724) and 9E10 (1:100; as above) antibodies, anti-FAZ10 (1:50; as above) and anti-ClpGM6 (1:200, a kind gift from Keith Gull). Alexa Fluor IgG secondary antibodies (Life Technologies) were used at 1:500. No nonspecific labelling was observed with these secondary antibodies alone (data not shown). Images were acquired (with identical exposures for samples with and without dox treatment) using a Leica DMI4000 B epifluorescence microscope, a Leica SP5 confocal microscope (LMMC – Multiuser Laboratory of Confocal Microscopy, FMRP/USP) or a Zeiss Axioskop2 microscope with Axiocam MRM camera (UoG).

### Electron microscopy

Transmission EM (TEM) of ultrathin sections of *T. brucei* PCF was performed as described by Moreira et al. (1991) and immunogold-EM was performed as described by Robinson et al. (1991) and Baqui et al. (1996). After cytoskeleton preparation, grids were incubated with mouse anti-FAZ10 (1:5; as above) and with rabbit anti-Myc antibodies (1:10, a gift from Ramanujan Hegde, University of Cambridge, UK) followed by secondary antibody goat anti-mouse 15 nm (1:40; EMS, cat.# 25133) and goat anti-rabbit 6 nm gold conjugates (1:40, EMS, cat.# 25104). After embedding and sectioning, the images were acquired at 120 kV using a Tecnai G2 electron microscope (LCMS, Laboratory of Cellular and Molecular Ultrastructure, FMRP/USP). Scanning EM (SEM) was performed as described elsewhere (Sant'Anna et al., 2005). Images were acquired using Jeol JSM-6610 LV microscope (LMME – Multiuser Electron Microscopy Laboratory, FMRP/USP).
